# The effects of seed from *Linum usitatissimum* cultivar with increased phenylpropanoid compounds and hydrolysable tannin in a high cholesterol-fed rabbit

**DOI:** 10.1186/s12944-018-0726-4

**Published:** 2018-04-10

**Authors:** Bożena Króliczewska, Dorota Miśta, Angelika Ziarnik, Magdalena Żuk, Jan Szopa, Ewa Pecka-Kiełb, Wojciech Zawadzki, Jarosław Króliczewski

**Affiliations:** 1 Department of Animal Physiology and Biostructure, Faculty of Veterinary Medicine, Wroclaw University of Environmental and Life Sciences, Norwida 31, 50-375 Wroclaw, Poland; 2 Sanitary and Epidemiological Inspection, Mickiewicza 24, 59-220 Legnica, Poland; 3 Department of Genetic Biochemistry, Faculty of Biotechnology, University of Wroclaw, Przybyszewskiego 63/77, 51-148 Wroclaw, Poland; 4 Department of Genetics, Plant Breeding and Seed Production, Faculty of Life Sciences and Technology, Wroclaw University of Environmental and Life Sciences, Norwida 31, 50-375 Wroclaw, Poland; 5 Department of Biology and Pharmaceutical Botany, Faculty of Pharmacy with Subfaculty of Laboratory Medicine, Medical University of Gdansk, Hallera 107, 80-416 Gdansk, Poland

**Keywords:** Flaxseed, Cholesterol, Diet, Lipid profile, GMO

## Abstract

**Background:**

Dietary fat is considered one of the most important factors associated with blood lipid metabolism and plays a significant role in the cause and prevention of atherosclerosis that has been widely accepted as an inflammatory disease of the vascular system. The aim of the present study was to evaluate the effect of genetically modified flaxseed (W86) rich in phenylpropanoid compounds and hydrolysable tannin in high cholesterol-induced atherosclerosis rabbit models compared to parental cultivar Linola.

**Methods:**

Twenty-Eight White New Zealand white rabbits aged 6 months were randomly divided into four groups, control group, high cholesterol group (10 g/kg), Linola flaxseed group (100 g/kg) and W86 flaxseed group (100 g/kg). The rabbits were fed a normal diet or a high cholesterol diet for 10 weeks. Levels of blood lipids, hematological values, total antioxidative status and superoxide dismutase activity in serum were determined. Moreover, body weight and feed intake were measured after sixth and tenth weeks. After each stage of the experiment atherogenic indexes (non-HDL-C/HDL-C, LDL-C/HDL-C, and atherogenic index of plasma) was calculated.

**Results:**

The intake of a dyslipidaemic diet negatively influenced lipid profile in rabbits at the 10 weeks of feeding. W86 flaxseed significantly decreased total cholesterol, LDL-C, VLDL-C and TG serum levels in cholesterolemic rabbits compared with parental Linola after 10 weeks. Atherogenic indexes decreased over time with a significant difference between the diets and they were the best for W86 flaxseed. Similarly, the experimental addition of W86 significantly decreased atherogenic predictors such as heterophil-to-lymphocyte ratio, platelet-to-lymphocyte ratio, and the mean platelet volume-to-lymphocyte ratio. In rabbits, W86 flaxseed increased the activity of superoxide dismutase and total antioxidative status compared to Linola.

**Conclusions:**

Results of the presented study suggest that the addition of W86 flaxseed alleviate serum lipid changes in high cholesterolemic diet-administered rabbits. W86 flaxseed significantly reduced atherogenic indexes, as compared with the Linola and indicate that W86 flaxseed more effectively red CVD risk factors during hypercholesterolemia. Moreover, the presented result suggested that W86 flaxseed can be a part of a heart-healthy and antiatherogenic diet for the human.

**Electronic supplementary material:**

The online version of this article (10.1186/s12944-018-0726-4) contains supplementary material, which is available to authorized users.

## Background

According to the World Health Organization, cardiovascular disease (CVD) is the leading cause of death worldwide, which represents 30% of all global deaths [1]. Dyslipidemia, including higher serum total cholesterol (TC) and low-density lipoprotein cholesterol (LDL-C) and lower high-density lipoprotein cholesterol (HDL-C), is a major risk factor for atherosclerosis. When atherosclerotic processes take hold in the arteries that supply blood to the heart, the condition becomes coronary artery disease (CAD). Moreover, inflammations and elevated platelet counts are associated with poor cardiovascular outcomes [2]. Dietary fat is considered one of the most important factors associated with blood lipid metabolism and plays a significant role in the cause and prevention of atherosclerosis [3]. In addition to hypercholesterolemia, hypertriglyceridemia may be another factor for diseases. Because cholesterol lowering is a major target for reducing CVD risk, dietary interventions to reduce TC and TG levels in individuals with borderline dyslipidemia and obesity without overall cardiovascular risk are becoming mandatory [3]. Large-scale clinical trials using statins have shown a significant reduction in cardiovascular events, mainly due to the lowering of plasma concentrations of LDL-C; however, the capacity of statins to prevent cardiovascular events are still limited to 30% to 40% of the patients treated even when intensive statin therapy is used [3, 4]. Nutraceuticals and functional food ingredients that are beneficial to vascular health may represent useful compounds that are able to reduce the overall cardiovascular risk induced by dyslipidaemia by acting parallel to statins or as an adjuvants [5].

Flax (*Linum usitatissimum* L.) is among the oldest plants with great significance in food production, healthcare, pharmaceutics, and industry as a source of fibre and oil. Moreover, flaxseed is one of the key sources of phytochemicals in the functional food area [6]. In addition to being one of the richest sources of *ω*-3 fatty acids and lignans, flaxseed is an excellent source of high-quality protein and soluble fibre and has considerable potential as a source of phenolic compounds [6]. It is established that polyunsaturated fatty acids (PUFA) like ω-3 (α-linolenic acid, ALA) and ω-6 (linoleic acid, LA) play important roles in human health and disease. Both are considered essential because they are not endogenously synthesized and must be obtained from the diet [7].

Due to the opposing effects of ω-3 and ω-6 fatty acids, a healthy diet should contain a balanced ω-6: ω-3 ratio. A balanced ω-6/ω-3 ratio 1–2/1 is one of the most important dietary. Human beings evolved eating a diet with a ω-6:ω-3 ratio of about 1:1 but modern diets exhibit LA: ALA ratios ranging between 15:1 to 30:1 [8, 9].

Excessive amounts of ω-6 and a very high ω-6/ω-3 ratio, promote the pathogenesis of many diseases, including cardiovascular disease, cancer, and inflammatory and autoimmune diseases, whereas increased level of ω-3 (a low ω-6/ω-3 ratio) exert suppressive effects [8, 10, 11]. *L. usitatissimum* cv. Linola seed exhibits an extremely high ω-6 to a ω-3 ratio (~ 31:1), so its health beneficial effect is rather limited [12]. However, new genetically modified *L. usitatissimum L.* namely W86 show the ~ 30-fold increase in α-linolenic acid level compared to the Linola cultivar [12]. Thus, the ratio of ω-6 to ω-3 in seed from W86 is about 1.3:1 [12], which is quite close to that recommended for the human diet [9]. Obviously, the bioavailability of ALA is dependent on the type of flax ingested [13].

Although, there are no data that suggest the LDL-C increases associated with some ω-3 fatty acid formulations lead to adverse outcomes, these increases in LDL-C may compromise the achievement of lipid targets; thus, there is a need for agents that can lower TG levels without increasing LDL-C levels [7] especially in individuals with high cholesterol levels.

Perhaps most importantly, there are some human conditions that cannot be adequately modelled by invertebrate or rodent species we chose rabbits. These animals are phylogenetically closer to primates than rodents and further offer a more diverse genetic background than inbred and outbred rodent strains [14]. The special characteristics of rabbit anatomy and physiology make it uniquely suitable for the study of hypercholesterolemia and atherosclerosis through diet which makes the model a better overall approximation to humans [15]. The rabbit model is highly reproducible with minimal variation between animals in a single laboratory and between laboratories.

Therefore, the purpose of presenting research was to identify the effect of flaxseed, a bioactive food compound on rabbits with high TC and TG levels. Particularly, this study aims to verify the clinical outcome of the change in the lipid profile due to the use of seeds from W86, a genetically modified *Linum usitatissimum* cv. Linola.

Moreover, the second objective was to determine if use of flax seeds with increase in α-linolenic acid level, and increased phenylpropanoid compounds, and hydrolysable tannin [12] has deleterious effects of red blood cells (RBCs), white blood cells (WBCs) and platelets and if these effects are different in normal and hypercholesterolemic rabbits.

## Methods

### Plant material

Flaxseed (cv. Linola) was obtained from the Flax and Hemp Collection of the Institute of Natural Fibres, Poland. Flaxseed W86 has been created with the aid of genetic engineering by agrotransformation of the Linola cultivar. In W86, down-regulation of the endogenous chalcone synthase gene (*CHS*) was achieved by overexpression of cDNA of the petunia homolog (*CHS*, EMBL/GenBank database acc. no. X04080) The details of plant transformation, selection, and transgenic plant analysis were described previously [12]. As a result of such overexpression, W86 plant demonstrates an increased phenylpropanoid contents resulting in a higher antioxidant capacity and accumulation of phenylpropanoid compounds as well as shows increase of hydrolysable tannin accumulation and reduction in lignin synthesis (Additional file 1: Table S1) [16]. Flaxseeds from control and transgenic plants were grown on a semi-technical scale in a field (Malbork, Poland) and harvested after 3 months of growth.

### Animals, diets and experimental protocol

Twenty-Eight White Giant rabbits aged 6 months ranging in weight from 3800 to 4400 g were used for the study. Housing requirements can be readily met for rabbits [17, 18]. After the adaptation period (rabbits were all fed on a normal diet), the rabbits were randomly divided into four groups of seven animals each. The groups were designated CTRL, CHOL, LIN, and W86. Group CTRL consisted of the control animals, which were fed a basal mixture for rabbits composed of dried grass, dried alfalfa, wheat bran, wheat grain, barley grain, corn, soybean meal, rape meal, rapeseed oil and vitamin/mineral premix (Granum Animal Nutrition, Poland). The chemical analyses of basal feed used in the experimental diets are presented in Additional file 1: Table S2. Group CHOL received the same basal diet with 1% cholesterol. Group LIN received the same diet as group CHOL supplemented with 100 g/kg of Linola flaxseed. Group W86 received the same diet as group CHOL but additionally supplemented with 100 g/kg of W86 flaxseed. The dose of flaxseed was calculated on the basis of flaxseed used in other studies [17]. The diets and fresh water were provided ad libitum and all manipulations were carried out between 09:00 and 16:00.

### Blood sampling

Blood samples were collected for analysis twice, after 6 weeks and at the end of the experiment (10 weeks). The blood and serum were processed in keeping with the standard procedures for the determination of each of the given parameters. Whole blood samples were collected from the lateral saphenous vein for hematological and biochemical analyses in a quiet, well-lit area to minimize stress. The rabbits were restrained in a towel, with the head covered, and held in lateral recumbency. The legs were extended and the fur that overlies the vein was delicately clipped. The skin was kept taut to immobilize the vein for blood collection. A 21-gauge needle fastened to a 5-mL syringe was used to collect the blood sample. To avoid hepatoma formation, gentle suction was followed by firm pressure after sampling.

### Analysis of hematological parameters

Red blood cell (RBC) count, haemoglobin (HGB) concentration, haematocrit (HCT), mean corpuscular volume (MCV), mean corpuscular haemoglobin (MCH), mean corpuscular haemoglobin concentration (MCHC), white blood cell (WBC) count and platelet (PLT) count, packed cell volume (PCV), red cell distribution width (RDW), and mean platelet volume (MPV) in rabbits fed experimental diets were determined by fully automated haematology analyser Horiba ABX (Horiba Medical) using commercially available diagnostic kits (Horiba ABX Diagnostics). Moreover, leucocytes counts were determined with particular counts of lymphocytes, monocytes, and heterophils.

For biochemical assays, blood serum was separated by centrifugation at 3000×g for 10 min. The separated serum was stored at − 20 °C for analysis.

### Biochemical analysis

Serum concentration of triglycerides (TG), total cholesterol (TC), and high-density lipoprotein cholesterol (HDL-C) were measured by enzyme-assay using commercial kits using ABX Pentra 400 (Horiba Medical). The fraction of plasma low-density cholesterol (LDL-C) was performed using LDL-C Direct CP kits from Horiba. VLDL-C in rabbits was calculated based on the formula VLDL Cholesterol = Total cholesterol - (HDL-C + LDL-C). For better expression of the relationship between triglycerides and HDL-C, atherogenic index of plasma (AIP) factor based on the ratio of the values of triglycerides to high-density lipoprotein (HDL-C) levels was calculated according to the following formula (AIP = Log [TGs]/ [HDL-C]) where both of them are measured in the plasma. AIP is used as a logarithmically transformed value because it produced better correlations and normal probability plots [19, 20]. Moreover, other atherogenic factors (AF) was calculated as the difference between non-HDL-C and HDL-C, LDL-C and HDL-C [21–25]. Total antioxidative status (TAS) and superoxide dismutase activities (SOD) were determined spectrophotometrically in serum using a kit from the Randox (Randox Laboratories Ltd., UK).

### Statistical analysis

Continuous variables are expressed as the mean ± standard deviation (SD). D’Agostino-Pearson normality tests were used to test for a normally distributed population [26]. Individual laboratory parameters were compared between groups by one-way analysis of variance (ANOVA) for feeding effects followed by the Bonferroni multiple-comparison test. Post hoc comparisons were made when appropriate. Multiple comparisons were performed only when ANOVA *P*-values were significant. The *P*-value was calculated under the null hypothesis that the samples were drawn from the same distribution. Statistical significance for ANOVA was accepted at a P-value less than 0.05. Diet effects and time effects (6 vs 10 weeks of diet), as well as diet × time, interactions were considered significant at *P* < 0.05. To assess the influence of dietary flaxseed, time of blood sampling and lipid contents of serum. Moreover, the heterophil-to-lymphocyte ratio (HLR), the platelet-to-lymphocyte ratio (PLR), and MPV to lymphocyte ratio (MPVLR) were calculated. All analyses were performed with Statistica 13.1 (Dell Inc., 2016) and R software (R Foundation Statistical Computing, Free Software Foundation, Inc.).

## Results

### Fatty acid analyses of flaxseed

The analysis of the fatty acid composition of the flaxseed from Linola and transgenic W86 demonstrated significant accumulation (*P* > 0.05) of ALA (~ 30 times more compared to maternal Linola, Table 1 and Additional file 1: Table S3). Furthermore, the W86 seed had significantly decreased percentages of LA (~ 37.7%).

**Table 1:** Fatty acid composition (percentage of fatty acids) of the flaxseed^a^

Fatty acid	Linola	W86
C14	0.083 ± 0.023	0.067 ± 0.012
C16	6.559 ± 0.588	5.847 ± 0.085
C16:1	0.142 ± 0.049	0.111 ± 0.001
C17	0.082 ± 0.015	0.076 ± 0.002
C17:1	0.044 ± 0.012	0.040 ± 0.002
C18	3.937 ± 0.969	4.737 ± 0.025
C18:1	19.449 ± 1.621	21.985 ± 0.022
C18:2 (LA)	66.945^A^ ± 2.592	41.709^B^ ± 1.171
C18:3 (ALA)	1.514^A^ ± 0.090	24.539^B^ ± 1.251
C20	0.155 ± 0.038	0.178 ± 0.003
C20:1	0.118 ± 0.007	0.127 ± 0.002
C20:2	0.061 ± 0.023	0.037 ± 0.001
C22	0.149 ± 0.045	0.159 ± 0.004
C20:5	0.141 ± 0.026	0.135 ± 0.004
Ʃ MUFA	19.753	22.263
Ʃ PUFA	68.660	66.420
Ʃ unsaturated	88.414	88.683
Ʃ saturated	10.965	11.064
n3/n6 ratio	0.022	0.588

^**a**^Mean ± SD values within the same row sharing a different superscript letter (A, B, C, etc.) are significantly different (*P* < 0.05)

In general terms, the flaxseed from Linola and W86 varieties had no significant (*P* > 0.05) different percentage of saturated and unsaturated fatty acids as well as PUFA and MUFA (Table 1). However, the n3/n6 fatty acid ratio is 0.588 in W86 flaxseed and is higher than in original Linola cultivar flaxseed (0.022).

### Effect of flaxseed on growth performance

The effects of dietary supplementations of flaxseed on growth performance and feed intake are summarized in Additional file 1: Table S4. There was no significant (*P* > 0.05) difference in final body weight of rabbits between all groups at the end of study (10 weeks). However, after 6 weeks of the diet, lower body weight was observed in CTRL and W86 groups (*P* < 0.05). Moreover, significant (*P* < 0.05) lower feed intake was reported in groups (LIN and W86) fed high cholesterol diet supplemented with flaxseed compared to both CTRL and CHOL groups. No significant changes in feed intake were observed after 10 weeks. So, on the other hand, after 10 weeks of breeding, the feed intake in the experimental groups was lower than the intake in 6 weeks: lower by 11.96% for the CTRL group and 11.97% for W86 group (*P* < 0.05). Significantly greater (*P* < 0.05) decline was observed in the group fed with cholesterol (16.13%) and in group LIN (14.69%).

### Haematological parameters

Cholesterol enrichment of red cell membranes associated with haemolytic anaemia occurs in rodent species fed diets enriched with cholesterol. Similar observations have been made in rabbits (5, 6). The results of the hematological analysis obtained after feeding rabbits a diet with the addition of two flaxseed varieties (Linola or its transgenic derivative W86) are expressed in Tables 2 and 3.

**Table 2:** Effects of atherogenic diet supplemented with flaxseed on hematological parameters of rabbits^*^

Parameters	CTRL	LIN	W86	CHOL
RBC (10^12^ L^− 1^)				
6 weeks	6.20 ± 0.64^A^	5.52 ± 0.72^B^	5.60 ± 0.46^B^	5.71 ± 0.95^B^
10 weeks	6.14 ± 0.94^A^	5.26 ± 1.36^B^	5.40 ± 0.55^B^	5.32 ± 0.86^B^
HGB (mmol L^− 1^)				
6 weeks	8.25 ± 0.81^A^	7.64 ± 0.63^B^	7.53 ± 0.7^B^	8.19 ± 1.27^A^
10 weeks	8.24 ± 1.19	7.48 ± 1.45	7.76 ± 0.52	7.7 ± 1.27
PCV (L L^− 1^)				
6 weeks	45.10 ± 4.23^A^	39.26 ± 3.68^B^	40.12 ± 1.78^B^	42.44 ± 6.63^Aa^
10 weeks	43.84 ± 6.93^A^	37.70 ± 8.21^B^	38.30 ± 3.80^B^	38.84 ± 6.03^Bb^
PLT (10^9^ L^− 1^)				
6 weeks	367.66 ± 57.20 ^Aa^	457.43 ± 214.11^Ba^	541.71 ± 158.50^C^	462.00 ± 133.00^Ba^
10 weeks	499.85 ± 214.61^Ab^	748.33 ± 194.69^Cb^	520.83 ± 146.53^A^	625.57 ± 133.41^Bb^
MCV (fL)				
6 weeks	73.00 ± 3.68	71.42 ± 3.91	72.71 ± 3.25	73.43 ± 1.81
10 weeks	71.57 ± 1.13	72.66 ± 5.43	74.17 ± 7.47	73.43 ± 5.00
MCH (fmol L^− 1^)				
6 weeks	21.41 ± 0.80	22.48 ± 2.37	22.80 ± 1.24	22.87 ± 0.67
10 weeks	21.67 ± 0.48	23.38 ± 2.21	24.37 ± 2.58	23.43 ± 1.72
MCHC (mmol L^− 1^)				
6 weeks	29.43 ± 0.85	31.43 ± 1.75	31.36 ± 0.82	31.09 ± 0.47
10 weeks	30.33 ± 0.616	32.10 ± 0.616	32.82 ± 1.08	31.97 ± 0.47
RDW (%)				
6 weeks	12.50 ± 0.86	11.75 ± 0.93	11.93 ± 0.77	12.47 ± 1.53
10 weeks	11.63 ± 0.28	11.05 ± 1.45	11.32 ± 1.69	11.09 ± 1.20
MPV (fL)				
6 weeks	6.04 ± 0.39^A^	5.72 ± 0.34^B^	5.37 ± 0.27^B^	5.39 ± 0.42^B^
10 weeks	5.86 ± 0.32^A^	5.53 ± 0.35^B^	5.27 ± 0.23^B^	5.27 ± 0.52^B^

^*^Mean ± SD values (*n* = 7) within the same row sharing a different superscript letter (A, B, C, etc.) are significantly different (*P* < 0.05) but values within the same column not sharing a common uppercase superscript letter (a, b, etc.) differ significantly (P < 0.05)

**Table 3:** Effects of atherogenic diet supplemented with flaxseed on WBC parameters of rabbits^*^

Parameters	CTRL	LIN	W86	CHOL
WBC (10^9^ L^−1^)				
6 weeks	8.77 ± 1.54^A^	13.01 ± 3.97^B^	13.77 ± 3.79^B^	12.51 ± 3.70^B^
10 weeks	9.59 ± 2.38^A^	13.22 ± 3.89^B^	14.80 ± 5.61^C^	13.69 ± 3.69^B^
Lymphocytes (10^9^ L^− 1^)				
6 weeks	5.35 ± 1.67^A^	7.10 ± 1.48^B^	8.67 ± 3.21^C^	8.60 ± 2.62^C^
10 weeks	5.50 ± 1.04^A^	7.11 ± 1.77^B^	10.50 ± 3.92^C^	8.80 ± 2.32^D^
Monocytes (10^9^ L^− 1^)				
6 weeks	0.15 ± 0.05	0.17 ± 0.08	0.20 ± 0.05	0.19 ± 0.07
10 weeks	0.13 ± 0.05^A^	0.20 ± 0.06^B^	0.22 ± 0.08^B^	0.19 ± 0.07^B^
Heterophils (10^9^ L^− 1^)				
6 weeks	2.50 ± 0.87^A^	3.10 ± 1.67^Ba^	3.40 ± 1.20^Ba^	3.35 ± 1.25^Ba^
10 weeks	2.70 ± 1.32^A^	4.00 ± 2.50^Bb^	4.20 ± 1.13^Bb^	4.79 ± 1.34^Bb^

^*^Mean ± SD values (n = 7) within the same row sharing a different superscript letter (A, B, C, etc.) are significantly different (P < 0.05) but values within the same column not sharing a common uppercase superscript letter (a, b) differ significantly (P < 0.05)

It followed from the present study that RBC, HGB, and PCV significantly (*P* < 0.05) decreased in groups fed high cholesterol diet with or without flaxseed (Table 2). The red cell indices (MCV, MCH, MCHC, and RDW) that are useful for elucidating anemias did not differ significantly (*P* > 0.05) between all groups (Table 2) [27]. Moreover, these values were within the normal range according to respected values [28, 29]. It is already known that platelets express and secrete inflammatory mediators (chemokines, sp-selectin, interleukin-1β, thromboxane, cytokines) and that elevated platelet counts were related to poor cardiovascular clinical outcomes [30–33]. The differences between groups and sequential changes in the platelet count and the mean platelet volume are summarized in Table 2. The 6 weeks platelet counts were similar in CHOL and LIN groups. However, in group W86 the counts were higher (*P* < 0.05) and were lower in CTRL group (*P* < 0.05) than in the other groups. The counts statistically increase (*P* < 0.05) in groups CTRL, and CHOL and LIN at 10 weeks as compared to 6 weeks. Surprisingly, the biggest change value was observed in group LIN (*P* < 0.001). The basal values for MPV were similar in all the groups except in CTRL groups where was the significantly highest (*P* < 0.05). The values at the end of 10 weeks period were similar in all the groups. We also did not note sequential, time-related changes in MPV values.

Rabbit leucocytic values are reported in Table 3. The normal WBC count in rabbits varies from 7.5 to 13.5 × 10^9^ L^− 1^ [29]. After 6 and 10 weeks of feeding, our results show a significant increase (*P* < 0.05) in WBC counts in LIN, W86 and CHOL groups compared to the CTRL group and slightly exceeded the normal value in group W86. Although, in contrast to the midterm result after 10 weeks, analyses showed a higher significant WBC increase in the W86 group. The sequential changes in WBC counts of the four groups are summarized in Table 3. At the end of 10 weeks, the values remained unaltered in all the groups except in group W86 where it increased as compared to the initial values (6 weeks).

The changes in the counts of lymphocytes, monocytes, and heterophils in the blood of the four experimental groups are summarized in Table 3. The total lymphocyte counts of all groups in both periods (6 and 10 weeks) differed markedly compared to CTRL group among treatments (*P* < 0.05) with a maximum value in group W86. At the end of 10 weeks, the values remained unchanged in groups CTRL, CHOL, and LIN but increased significantly (*P* < 0.05) in group W86 as compared to the 6 weeks values. On the other hand, the total monocytes count showed a non-significant (*P* > 0.05) level among all groups after 6 weeks, but at the end of experiment slightly but significant (*P* < 0.05) increase was observed in all groups compared to CTRL group. At the end of experimental periods, the values remained unaltered in all the groups as compared to first blood sampling. This study demonstrated that heterophils counts after 6 and 10 weeks were significantly (*P* < 0.05) higher in all groups compared to CTRL. Moreover, there were no differences between LIN, W86 and CHOL groups in both periods. It has also been demonstrated that heterophil counts significantly changes over time in all groups except in CTRL group.

Thrombocytosis and inflammation are vital elements in the pathogenesis of atherosclerosis. The platelet-to-lymphocyte ratio (PLR) is a novel biomarker that combines these parameters and has been shown to be associated with CVD. Moreover, previous research had also revealed a significant association between coronary heart risk and PLR and neutrophil-lymphocyte ratio NLR (HLR ratio for rabbit) [34, 35]. Rabbit has a heterophil instead of a neutrophil and rabbits heterophil functions like the neutrophil of other species and is only histochemically distinct [36].

**Fig. 1:**
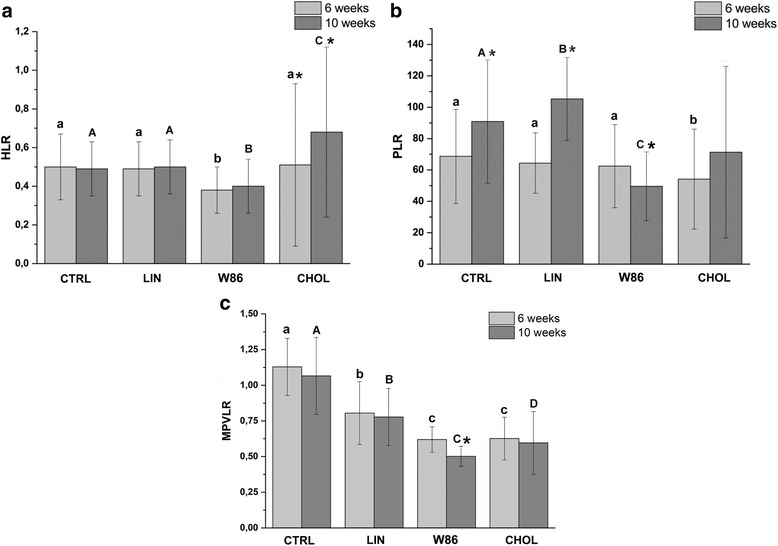
Predictors of atherosclerosis and acute coronary syndrome. Comparison of HLR (**a**) PLR (**b**) and MPVLR ratio (**c**) between groups. Error bars represent standard deviations. Results are expressed as the means ± SD; statistically different values are marked with lower case letters (6 weeks) or capital letters (10 weeks) when a significant interaction was observed. Significant changes in the same group between 6 and 10 weeks (*p* < 0.05) are marked with an asterisk

In the current study, after 6 weeks, the HLR values was 0.5 ± 0.17 in the CTRL group, 0.49 ± 0.14 in the LIN group, 0.38 ± 0.12 in the W86 group, and 0.51 ± 0.42 in CHOL group (Fig. 1a). There was no statistically significant difference between the two groups (CTRL and LIN) in respect of the HLR values after both 6 (see above) and 10 weeks (0.49 ± 0.14 and 0.5 ± 0.14 respectively). However, a significant reduction of HLR occurred in a group of rabbits fed the atherogenic diet supplemented with W86 flaxseed (0.38 ± 0.12 and 0.4 ± 0.14) (Fig. 1a). The CHOL group showed statistically significantly (*P* < 0.05) higher HLR values (0.68 ± 0.44) compared to all groups after the 10 weeks period. In addition, this recent study (Fig. 1a) indicates a significant increase (*P* < 0.05) of HLR ratio in CHOL group over time.

In a next step, a PLR value in all groups was calculated. The changes at both the 6 and 10 weeks in the PLR ratio in the four experimental groups are shown in Fig. 1b. The PLR values at 6 weeks in groups CTRL, LIN, W86, and CHOL were respectively 68.72 ± 29.96, 64.43 ± 19.28, 62.48 ± 26.57, and 54.20 ± 32.89. There was no difference in the PLR ration between the groups at the end of 6 weeks. The PLR values changed in groups CTRL, LIN, and CHOL but decreased in group W86 with respect to 6 weeks and were respectively 90.88 ± 39.27, 105.25 ± 26.47, 49.60 ± 21.94, and 71.20 ± 54.75. In second sampling periods, the PLR ratio in group W86 was significantly lower than in other groups (Fig. 1b). Moreover, only in this group, we observed a significant decrease in time (*P* < 0.05). In contrast, PLR ratio significantly increases (*P* < 0.05) in groups CTRL and LIN. We did not observe significant differences in PLR between CHOL and others group in the 10th week due to high standard deviation value in CHOL group.

The MPVLR data was presented in Fig. 1c. The most important observation is that in group W86 MPVRL significantly (*P* < 0.05) decreased over time during the study (from 0.61 ± 0.09 to 0.50 ± 0.07). In the other groups, no significant changes were noted between 6 and 10 weeks. Moreover, lowest MPVRL was also shown in group W86 at the 10th week of the experiment.

### Biochemical parameters

The results of the biochemical blood analysis are shown in Tables 4 and 5. The concentrations of serum TC were changed in groups LIN, W86, and CHOL during the 6 and 10 weeks feeding period compared to CTRL group and was statistically significant (P < 0.05). Similar results were obtained for LDL-C and TG concentrations. Furthermore, the results show that the rabbits from groups LIN, W86 and CHOL were hypercholesterolemic. The inclusion of W86 variety of flaxseed into a diet of rabbits had statistically significant (*P* < 0.05) effects on the serum lipid compared to groups LIN and CHOL. Generally, we have observed that the TC and LDL-C concentration was significantly lower (P < 0.05) in rabbits supplemented with flaxseed from W86 variety compared to rabbits from LIN and CHOL groups. Similar results were obtained in the TG concentration of the hypercholesterolemic rabbits. Also, in this case, TG concentration was significantly lower (P < 0.05) compared to LIN and CHOL groups. Moreover, after 6 weeks of the experiment, the TG concentration was similar to TG concentration in CTRL group. There was no significant difference between the high cholesterol-fed groups in terms of HDL-C concentration. Moreover, these results demonstrate time stable (Table 4) levels of HDL-C in all groups in contrast to CTRL. Comparing of average serum lipids contents in rabbits supplemented with flaxseed from both varieties (maternal Linola and its W86 derivative) we have shown that W86 flaxseed possessed higher antiatherogenic potential.

**Table 4:** Effect of high-fat diet and flaxseed on lipid profile of controls and experimental rabbit groups^*^

Parameters	CTRL	LIN	W86	CHOL
TC (mmol L^−1^)				
6 weeks	0.82 **±** 0.18^A^	16.40 **±** 3.27^B^	11.87 **±** 2.28^C^	12.50 **±** 5.29^C^
10 weeks	0.79 **±** 0.30^A^	15.66 **±** 5.46^B^	12.17 **±** 3.38^C^	13.53 **±** 4.39^D^
non-HDL-C (mmol L^− 1^)				
6 weeks	0.40 **±** 0.22^Aa^	15.79 **±** 5.40^B^	11.27 **±** 2.33^Ca^	11.81 **±** 3.45^C^
10 weeks	0.24 **±** 0.15^Ab^	15.26 **±** 6.28^B^	10.35 **±** 2.29^Cb^	12.87 **±** 4.92^D^
LDL-C (mmol L^− 1^)				
6 weeks	0.19 **±** 0.05^A^	8.65 **±** 3.59^B^	5.93 **±** 1.36^C^	6.88 **±** 2.06^D^
10 weeks	0.23 **±** 0.09^A^	8.21 **±** 5.10^B^	6.27 **±** 2.13^C^	7.01 **±** 2.80^C^
HDL-C (mmol L^−1^)				
6 weeks	0.41 **±** 0.14^A^	0.61 **±** 0.16^B^	0.60 **±** 0.10^B^	0.68 **±** 0.26^B^
10 weeks	0.56 **±** 0.24^A^	0.59 **±** 0.08^A^	0.57 **±** 0.21^A^	0.67 **±** 0.21^B^
VLDL-C (mmol L^− 1^)				
6 weeks	0.21 **±** 0.22^A^	7.13 **±** 2.22^B^	4.67 **±** 2.36^C^	4.93 **±** 1.59^C^
10 weeks	0.15 **±** 0.09^A^	5.97 **±** 2.92^B^	3.96 **±** 2.79^C^	5.36 **±** 2.30^B^
TG (mmol L^−1^)				
6 weeks	0.74 **±** 0.13^Aa^	1.02 **±** 2.41^B^	0.74 **±** 0.25^A^	1.48 **±** 1.84^B^
10 weeks	0.57 **±** 0.16^Ab^	1.03 **±** 0.27^B^	0.81 **±** 0.21^C^	1.31 **±** 0.29^D^

^*^Mean ± SD values within the same row sharing a different superscript letter (A, B, C, etc.) are significantly different (P < 0.05) but values within the same column not sharing a common uppercase superscript letter (a, b) differ significantly (P < 0.05)

**Table 5:** Effect of high-fat diet and flaxseed on TAS and SOD values in the studied groups^*^

Parameters	CTRL	LIN	W86	CHOL
SOD (U mL^−1^)				
6 weeks	269.29 ± 80.48^Aa^	246.14 ± 68.34^Ba^	240.14 ± 54.09^Ba^	258.57 ± 87.20^Ca^
10 weeks	179.67 ± 47.33^Ab^	152.34 ± 58.61^Bb^	163.5 ± 60.83^Cb^	139.00 ± 32.55^Bb^
TAS (mmol L^− 1^)				
6 weeks	1.77 ± 0.04^a^	1.84 ± 0.08^a^	1.80 ± 0.07^a^	1.79 ± 0.11^a^
10 weeks	1.63 ± 0.09^Ab^	0.97 ± 0.75^Bb^	1.36 ± 0.34^Cb^	1.30 ± 0.27^Db^

^*^Mean ± SD values within the same row sharing a different superscript letter (A, B, C, etc.) are significantly different (P < 0.05) but values within the same column not sharing a common uppercase superscript letter (a, b) differ significantly (P < 0.05)

Importantly observation was that in groups W86 and LIN significant decrease in VLDL-C contents were demonstrated after 10 days of feeding in contrast to the CHOL group (Table. 1). However, it was shown that better effects were observed in the W86 group (*P* < 0.05), since in group LIN this content was still higher than in group CHOL.

**Fig. 2:**
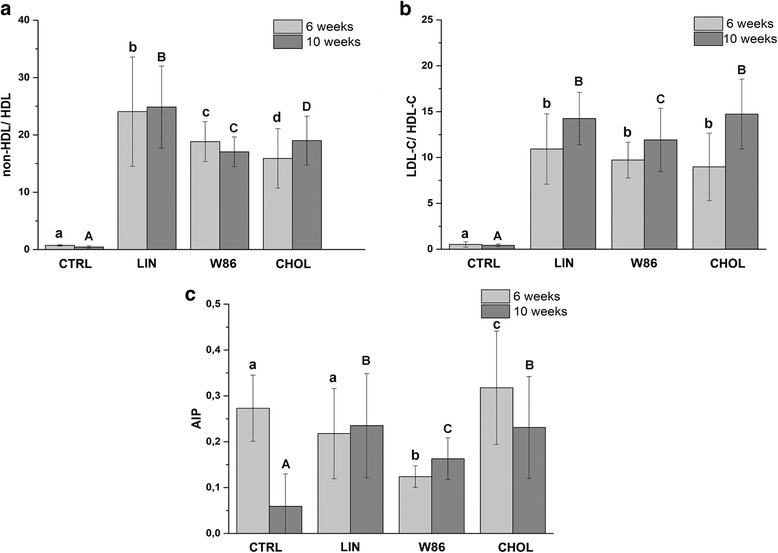
Effects of flaxseed on atherogenic indexes in rabbits fed hypercholesterolemic diet. **a** non-HDL-C/HDL-C, **b** LDL-C/HDL-C ratios and **c** AIP index of rabbits fed control diet (CTRL), high cholesterol diet (CHOL) and high cholesterol diet supplemented with flaxseed of Linola (LIN) or Linola W86 derivative (W86). Results are expressed as the means ± SD; statistically different values are marked with lower case letters (6 weeks) or capital letters when a significant interaction in 10 weeks was observed. Significant changes in the same group between 6 and 10 weeks (*p* < 0.05) are marked with an asterisk

The effects of dietary Linola and W86 derivative flaxseed in a rabbit model, summarizing the atherogenic lipid ratios are presented in Fig. 2. Based on the data presented in Table 4, mean atherogenic lipid ratios: non-HDL-C: HDL-C (Fig. 2a) and LDL-C: HDL-C (Fig. 2b) were calculated. Furthermore, AIP index (Fig. 2c) and non-HDL-C content were also calculated (Table 4) [21–23, 25]. The non-HDL-C cholesterol is the sum of all the lipoproteins which contribute towards the development of atherosclerosis (narrowing of the arteries) and is thought to be a better predictor of cardiovascular disease (CVD) than using LDL-C alone (Table 4). In the present study, non-HDL-C levels remained unchanged throughout a period of observation in groups LIN and CHOL but slightly decreased (*P* > 0.061) at 10 weeks in group W86 as compared to 6 weeks results. Moreover, at the end of experiments (10 weeks) rabbits fed diet supplemented with W86 flaxseed shown significantly lower “non-HDL-C” level comparing to rabbits from CHOL and LIN groups. W86 treatment affected the derived non-HDL-C/HDL-C (Fig. 2a) and LDL-C/HDL-C ratios (Fig. 2b), and the AIP index (Fig. 2c). The animals of the W86 group showed significantly lower ratios of non-HDL-C/HDL-C, LDL-C/HDL-C, and AIP index than the rabbits in the other groups exclude CTRL group.

Linola flaxseed (group LIN) did not positively affect the AIP index nor LDL-C/HDL-C or non-HDL-C/HDL-C ratios. The non-HDL-C/HDL-C ratio was significantly higher (*P* < 0.05) than in CHOL group. Furthermore, LDL-C/HDL-C ratio and AIP index were similar in group LIN and CHOL.

The effects of high cholesterol diet, with and without dietary flaxseed, on SOD and TAS levels, are summarized in Table 5.

To evaluate the antioxidant effect of flaxseed on vascular in our experiment, we detected the serum levels of SOD and TAS. The results of the analysis were listed in Table 5. We can draw from the Table 5 that, compared to the CTRL group, serum SOD levels of rabbits in the others groups dropped greatly (*P* < 0.05), after high cholesterol diet in 6 and 10 weeks. The levels of SOD decrease (*P* < 0.05) between 6 and 10 weeks 93.80, 76.64 and 119.57 in groups, LIN, W86, and CHOL, respectively. However, decreases in the W86 group were significantly smaller (*P* < 0.05) than those in the LIN and CHOL groups. There were statistically significant differences (*P* < 0.05) in TAS between groups CTRL, LIN, W86 and CHOL after 10 weeks. As was mentioned above, in groups LIN and CHOL, rabbits had highest decreased SOD activities and TAS. It should be stressed that those were rabbits having the highest TC and LDL-C concentrations. In the same groups, higher LDL-C/HDL-C ratios and AIP index was observed.

## Discussion

Naturally derived dietary supplements capable of modifying dyslipidemias are becoming more popular and attractive to patients. Among them are *Linum usitatissimum* L., the richest known source of PUFA, and lignans [6]. Most commonly used forms include flaxseed, flaxseed flour and flaxseed oil [37–39]. Moreover, nutraceuticals are an interesting option for patients with dyslipidemia owing to their safety, tolerability, and ability to lower plasma lipid levels and play a peculiar role in ameliorating human dyslipidemia [5, 40]. These roles of nutraceuticals were detailed reviewed by Scicchitano, et al. [5]. In their review, the authors focused on the mechanism of action of nutraceuticals and functional food ingredients on lipids and their role in the management of lipid disorders.

The aim of this study was to assess the impact of W86 flaxseed with increased phenylpropanoid compounds and hydrolysable tannin on hematological parameters and atherogenic indexes in a hypercholesterolemic diet based on the results in alterations in the lipid profile. Moreover, we investigated the effect of short-term use of flaxseed, with special emphasis on variety W86, in rabbits on the hemopoietic and lymphopoietic system.

Our results show that exposure of rabbits to W86 flaxseed during the full fattening period did not have a major impact on the body weight and feed intake. However, the feed intake in the experimental groups was lower at the end of this period than in 6 weeks and significantly greater (*P* < 0.05) decline was observed in the LIN group than in W86 group during the experiment.

In spite of the well-known variability of hematological parameters in rabbits with regard to breed-related and individual differences, the measured values of RBC, HGB, and PCV count in this research occur within the range of reference physiologic values for rabbits [29]. Although, the values of the RBC in blood in three groups (CHOL, LIN, and W86), were near the bottom level of the range stipulated for adult New Zealand white rabbits (4–7.2 × 10^12^ L^− 1^), in our opinion the RBC, HGB and PCV values just like MCV, MCH, MCHC, and RDW were physiological for the age of the tested rabbits [28, 29]. Moreover, demonstrated results suggest that use of W86 flaxseed does not have deleterious effects on the hemopoietic system.

Studies on HLR, PLR, and MPV have grown recently following the discovery of their immense values in prediction and prognosis of many medical conditions and are potent markers of inflammation which underlies the basic pathologies of various diseases [41–43]. Furthermore, the chronic inflammatory response is a critical element in the pathogenesis of atherosclerosis and is associated with the production of lymphocytes and PLT. Elevated activated platelets count promotes atherothrombosis, atherosclerosis formation, progression, and destabilization of atherosclerotic plaques, and are associated with an incidence of CAD [44, 45]. The present results demonstrated that there was an increase in platelet counts in both CHOL and LIN groups. However, PLT values in rabbits supplemented with W86 flaxseed at week 10 were the same as in the CTRL group.

Cardiovascular events are primarily associated with a high circulating platelet count and low blood lymphocytes. Moreover, the PLR has come into use recently as an indicator of the balance between inflammation and thrombosis and act as an effective biomarker to predict CVD risk [44]. Furthermore, PLR gives an idea about both the aggregation and inflammation pathways and it may be more valuable than either platelet or lymphocyte count alone in the prediction of coronary atherosclerotic burden [46]. High PLR values are associated with cardiovascular diseases and adverse outcomes [47]. It has been reported that NLR (analogue of HLR) is a marker for progression of atherosclerosis in coronary artery disease and a marker for possible cardiac events and mortality in patients with stable coronary artery disease [34, 48]. The present results demonstrated that W86 flaxseed significantly decreased PLR and HRL ratios in rabbits fed high cholesterol diet. Moreover, W86 flaxseed significantly decreased MPVLR in contrast to parental Linola flaxseed (group LIN). Several studies reviewed by Nording, et al. [32] support the roles of platelets in chronic low-grade inflammation driving atherosclerosis. In our study, we found that W86 flaxseed positively influenced on the PLT count. The PLT of the high cholesterol-W86 fed groups was statistically significantly lower than that of the CHOL, and LIN groups and similar to CTRL. This result suggests that W86 flaxseed with increased phenylpropanoid compounds, hydrolysable tannin, and the well balanced ω-6/ω-3 ratio can prevent thrombocytosis and eventually atherosclerotic lesions.

The rabbit fed with typical laboratory chow diets contain typical plasma cholesterol concentrations in the range of ~ 0.78–0.91 mmol L^− 1^ as we showed in CTRL group [29, 49]. The rabbit rapidly develops severe hypercholesterolemia leading to premature atherosclerosis in response to dietary manipulation. Diets containing high cholesterol amounts (0.3–0.5%) have demonstrated that the hypercholesterolemic rabbit can develop complex, advanced lesions that more closely resemble those found in humans [50, 51]. In this study, a high-cholesterol diet resulted in hypercholesterolemia in rabbits, and blood TG and TC levels were increased compared to that measured in CTRL counterparts group.

The increased use of ALA is a powerful example of one such nutritional strategy that may produce significant cardiovascular benefits. Based on the results of clinical trials, epidemiological investigations and experimental studies, ingestion of ALA has been suggested to have a positive impact on CVD. Increased linoleic intake has been shown to promote platelet aggregation and oxidation of LDL. Renaud, et al. [52] demonstrated that α-linolenic acid has antithrombotic properties and may also be antiarrhythmic. Moreover, α-linolenic acid is a precursor of other omega-3 fatty acids such as eicosapentaenoic acid (EPA) and docosahexaenoic acid (DHA), which may have independent beneficial effects [53]. Harper, et al. [54] showed that patients which received linseed capsules demonstrated a 25% increase in the plasma values of docosapentaenoic acid (DPA), a cardioprotective fatty acid in the linseed group while the olive oil group showed no changes.

The HDL-C is recently gaining sample scientific attention due to its antiatherogenic properties providing cardioprotection. The relationship between HDL-C and cardiovascular diseases is primarily due to cholesterol efflux facilitated by apolipoprotein A1 during the process called the reverse cholesterol transport and HDL-C acceptors or by diffusion leads to HDL-C mobilization from cell membrane to hepatic storage. In addition to this, HDL-C exerts antithrombotic activity by preventing platelet aggregation [55]. Furthermore, HDL-C prevents oxidative modification of LDL and blocks the pro-inflammatory effects of oxidized LDL [56]. However, Prasad, et al. [57] showed that HDL-C can also have proinflammatory effects. In the research of Bierenbaum, et al. [58] hyperglycaemic patients who received linseed together with vitamin E daily for 3 months exhibited a significant reduction in serum TC, LDL-C and serum lipid oxidation products while HDL-C did not change at all. In this trial, statistically insignificant changes (*P* = 0.09) between group CTRL, LIN, and W86 were observed in HDL-C levels in rabbits at week 10 in contrast to group CHOL. However, this statistically significant increase should be rather considered together with generally high TC and lipoprotein content in rabbit fed cholesterol without flaxseed.

Peng, et al. [59] stated in their review, that a large number of studies have shown that hypertriglyceridemia contributes to the development and progression of atherosclerosis. However, the proatherogenic mechanism of hypertriglyceridemia seems rather complicated and needs to be further investigated. Based on current knowledge and the evidence of clinical studies, controlling and lowering plasma TG levels is one of the important measures to further reduce the residual risk of CVD events in atherosclerotic CAD patients. Stuglin and Prasad [60] suggested that the linseed increased the TG level while the others demonstrated the reduction in TG concentration induced by the linseed [61]. In our study, TG concentration was lowered through both linseed supplementation in contrast to CHOL group. However, herein we show that W86 flaxseed showed more beneficial effect than flaxseed from the parental variety. What is more important is that W86 flaxseed in contrast to parental variety reduced not only TG but also TC level in hypercholesterolemic rabbits (*P* = 0.034). This result is opposed to the previous observation of Dupasquier, et al. [62]. These authors showed that flaxseed in the diet reduced serum TG but had no effect on serum TC in rabbits. Moreover, Prasad [63] reported an increase in serum TC and no change in serum TG in rabbits on a flaxseed diet as we have shown for Linola flaxseed (group LIN).

The non-HDL-C incorporates the harmful elements of the lipoprotein profile to include TG rich remnant particles from VLDL and IDL, as well as LDL-cholesterol. It is also better for monitoring response to treatments such as lifestyle changes and medication. The non-HDL-C has been recommended as the target for lipid modification in guidelines by the National Institute for Health and Care Excellence. Moreover, the non-HDL-C includes all ApoB containing lipoproteins and for this reason, the non-HDL-C/HDL-C ratio provides cardiovascular risk stratification similar to the ApoB/ApoA1 ratio in diabetics as a comparison with reference lipid marker [64]. Herein, we provided evidence for the non-HDL-C, VLDL-C and LDL-C lowering properties of W86 flaxseed in contrast to Linola seeds.

Atherogenic indexes are considered as a better indicator of coronary heart disease risk than individual lipoprotein concentration. The results of the present study showed that W86 flaxseed also caused a significant reduction in all calculated atherogenic indexes. Furthermore, this beneficial effect is more valuable than was observed for Linola.

The high-fat diet also causes oxidative stress, thus, increases oxidation of LDL which plays a key role in the genesis of atherosclerosis but antioxidants such as flavonoids are known to effectively prevent this kind of damage [65]. The presence of the strong antioxidant in the flaxseed such as anthocyanins, flavones, and proanthocyanins may also offer additional benefit against oxidative stress caused by high cholesterol. Flaxseed from W86 cultivar contains more lignans and tannins than its parental Linola (Additional file 1: Table S1). The most remarkable lignan is secoisolariciresinol diglucoside (SDG), a very potent antioxidant [66]. The beneficial effect of flax lignan complex was reviewed by Prasad [67]. Moreover, Gato, et al. [68] indicate that tannin is a useful food material for treating hypercholesterolemia while Si, et al. [69] reported antioxidant activity of tannins. In the present study, we used SOD radical scavenging properties to examine the antioxidant blood activity. Nevertheless, another recently developed method measuring the total antioxidant status (TAS) provides the evaluation efficiency of the biological interactions between individual antioxidant species such as antioxidant enzymes and low-molecular-weight antioxidants and also a measure of the capacity of biological systems to withstand oxidative attack [70]. Statistical analysis showed significant differences among study groups in the levels of TAS and SOD between 6 and 10 weeks and in all groups both parameters decreased after 10 weeks. No statistically significant differences were found in serum TAS values among groups after 6 weeks. In group LIN we demonstrated a drastic decrease of TAS at 10 weeks in contrast to a W86 group. The present findings are in agreement with previous observations showed that flaxseed oil had no effect on the activity of SOD or flaxseed oil increased the levels of lipid peroxidation and that this effect was associated with the reduction in SOD activity [71, 72]. The research data suggest that antioxidant activity of flaxseed seems to be due to the SDG present in these components [73]. The findings of the present investigation confirm those reports and expand previous observations by demonstrating that W86 flaxseed richer in phenylpropanoid compounds, flavonoids and hydrolysable tannin than Linola can better slow the progression of atherosclerosis by reducing oxidative stress. Additionally, the amount of ALA that is present in the diet is very small, and dietary supplementation is needed whenever increased oxidative stress in the body leads to clinically significant consumption of endogenous antioxidants.

Moreover, McCullough, et al. [74] demonstrated that ALA content of flaxseed is associated with an induction of adipose leptin expression encoded by the “*obese”* gene. Leptin protein levels were elevated in animals taking diet supplemented with 10% flaxseed. Changes in leptin expression were strongly and positively correlated with adipose ALA levels and inversely correlated with risk of atherosclerosis. W86 flaxseed contains ~ 30-fold excess of ALA than parental Linola and may be a good supplement to obesity prevention but this suggestion should be further investigated.

### Limitations of the study

Our study has some limitations including the routine measurement of HDL-C, without HDL-C subclass assessment, we cannot discriminate between atheroprotective vs. dysfunctional, less atheroprotective or even atherogenic particles. Lack of other established inflammatory markers, such as C-reactive protein, and tumor necrosis factor-a, is another limitation of the study. The findings of the study should be generalized across CAD and atherosclerotic patients in clinical practice. Moreover, a molecular study needs to be performed to more accurately explain the beneficial effect of genetically modified flax W86. Another limitation of this study is that since the CTRL group was not administered any flaxseed supplementation.

## Conclusions

The present study suggested that W86 flaxseed from genetically modified Linola affect lipid metabolism and lower atherogenic indexes in rabbits. Accurate documentation of the therapeutic effects of W86 flaxseed and its components that contribute uniquely to disease prevention health protection and as a deterrent to degenerative diseases will increase its potential for use as a functional food and food ingredient. Hence, we can conclude that W86 flaxseed provides a safe and relatively inexpensive alternative for reducing cholesterol and thereby the risk of CVD and be a good supplement in atherosclerosis and obesity prevention. Moreover, we believe that this result supports the hypothesis of about the use of nutraceuticals in primary cardiovascular prevention, protocols to reduce the overall burden of cardiovascular disease morbidity and mortality as was suggested by Scicchitano, et al. [5]. Nevertheless, further studies are needed to implement the actual findings associated with this hypothesis.

## Additional file

Additional file 1: **Table S1.** Biochemical composition of W86 seeds used in this study (mg g^− 1^) in dry weight. The results are reported as a mean ± SD. (*n* = 4). **Table S2.** The chemical analyses of basal diet. **Table S3.** Fatty acid composition of the flaxseed used in this study (g/kg FW^*^). **Table S4.** Average values of body weight and feed intake during the experimental period. The results are reported as a mean ± SD, (*n* = 7). (DOCX 38 kb)

## Abbreviations

AIP: Atherogenic index of plasma
ALA: α-linolenic acid
CAD: Coronary artery disease
CVD: Cardiovascular disease
HCT: Hematocrit
HDL-C: High density lipoprotein cholesterol
HGB: Hemoglobin concentration
HLR: Heterophil-to-lymphocyte ratio
LA: Linoleic acid
LDL-C: Low density lipoprotein cholesterol
MCH: Mean corpuscular hemoglobin
MCHC: Mean corpuscular hemoglobin concentration
MCV: Mean corpuscular volume
MPV: Mean platelet volume
MPVLR: MPV to lymphocyte ratio
MUFA: Monounsaturated fatty acids
PCV: Packed cell volume
PLR: Platelet-to-lymphocyte ratio
PLT: Platelets
PUFA: Polyunsaturated fatty acids
RBC: Red blood cells
RDW: Red cell distribution width
SD: Standard deviation
SOD: Superoxide dismutase
TAS: Total antioxidative status
TC: Total cholesterol
TG: Triglyceride
WBC: White blood cells
