# Supplementary material for: The effects of seed from Linum usitatissimum cultivar with increased phenylpropanoid compounds and hydrolysable tannin in a high cholesterol-fed rabbit
Source: Lipids Health Dis. 2018 Apr 10;17:76. doi: 10.1186/s12944-018-0726-4 (PMC5891892; doi:10.1186/s12944-018-0726-4)
Supplement: Supplementary file 1 — Table S1. Biochemical composition of W86 seeds used in this study (mg g− 1) in dry weight. The results are reported as a mean ± SD. (n = 4). Table S2. The chemical analyses of basal diet. Table S3. Fatty acid composition of the flaxseed used in this study (g/kg FW*). Table S4. Average values of body weight and feed intake during the experimental period. The results are reported as a mean ± SD, (n = 7). (DOCX 38 kb) [file 12944_2018_726_MOESM1_ESM.docx]

# Supplementary Information

# The effects of seed from *Linum usitatissimum* cultivar with increased phenylpropanoid compounds and hydrolysable tannin in a high cholesterol-fed rabbit.

Bożena Króliczewska^a1^, Dorota Miśta^a1^, Angelika Ziarnik^b^, Magdalena Żuk^c^, Jan Szopa^c^, Ewa Pecka-Kiełb^a^, Wojciech Zawadzki^a^, Jarosław Króliczewski^d 🖂^

^a^Department of Animal Physiology and Biostructure, Faculty of Veterinary Medicine, Wroclaw University of Environmental and Life Sciences, Wroclaw, Poland.

^b^Sanitary and Epidemiological Inspection, Legnica, Poland.

^c^Department of Genetic Biochemistry, Faculty of Biotechnology, University of Wroclaw, Wroclaw, Poland.

^d^Department of Chemical Biology, Faculty of Biotechnology, University of Wroclaw, Wroclaw, Poland.

^🖂^Correspondence should be addressed to: JK, e-mail: [jakrol@windowslive.com](mailto:jakrol@windowslive.com).

**Table S1.** **Biochemical composition of W86 seeds used in this study (mg g^-1^) in dry weight.** The results are reported as a mean ± SD. (n=4)

| **Items** | **Linola** | **W86** |
| --- | --- | --- |
| Anthocyanins | 0.031 ± 0.004 | 0.069 ± 0.009 |
| Flavones | 0.00088 ± 0.00007 | 0.00094 ± 0.00010 |
| SDG* | 179.20 ± 4.90 | 193.27 ± 3.43 |
| Proanthocyanins | 0.31 ± 0.02 | 0.34 ± 0.09 |
| Hydrolysable tannins | 0.57 ± 0.12 | 2.69 ± 0.38 |

***** secoisolariciresinol diglucoside

**Table S2. The chemical analyses of basal diet.**

| **Items** | **g/kg** |
| --- | --- |
| Crude protein | 160.0 |
| Crude lipid | 36.0 |
| α-linolenic acid, (ALA) | 0.9 |
| linoleic acid, (LA) | 4.6 |
| Crude fibre | 115.0 |
| Ash | 90.0 |
| Sugar | 74.0 |
| DNFE (digestible nitrogen-free extract) | 470.6 |
| Phosphorus | 7.6 |
| Calcium | 10.0 |
| Sodium | 2.0 |
| Methionine | 4.0 |
| Lysine | 8.0 |
| Selenium (mg) | 0.3 |
| Net energy (MJ/kg) | 14.60 |
| Metabolizable energy (MJ/kg) | 10.34 |
| Digestible energy (MJ/kg) | 7.8 |

**Table S3. Fatty acid composition of the flaxseed used in this study (g/kg FW^*^)**

| **Fatty acid** | **Linola** | **W86** |
| --- | --- | --- |
| 16:0 | 11.33 | 13.60 |
| 16:1 | 0.16 | 0.22 |
| 16:2 | 0.12 | 0.17 |
| 16:3 | 0.10 | 0.10 |
| 18:0 | 6.38 | 7.63 |
| 18:1 | 32.57^A^ | 40.05^B^ |
| **18:2 (LA)** | **158.57** | **153.91** |
| **18:3 (ALA)** | **3.64**^A^ | **105.70**^B^ |
| 20:0 | 0.26 | 0.26 |
| 20:1 | 0.19 | 0.19 |
| 22:0 | 0.12 | 0.14 |
| 22:1 | 0.10 | 0.18 |
| 24:0 | 0.03 | 0.06 |
| **Total** | **213.57**^A^ | **322.22**^B^ |
| saturated | 18.13 | 21.70 |
| unsaturated | 195.44^A^ | 300.52^B^ |
| PUFA | 162.43^A^ | 259.89^B^ |
| MUFA | 33.02^A^ | 40.64^B^ |

^*^Fresh weight. Values within each row not sharing a common superscript letter (A. B. C. etc.) are significantly different (P < 0.05).

**Table S4. Average values of body weight and feed intake during the experimental period.** The results are reported as a mean ± SD, (n=7).

| **Parameters** | **Time** | **Experimental group** | | | |
| --- | --- | --- | --- | --- | --- |
|  |  | **CTRL** | **CHOL** | **LIN** | **W86** |
| Initial body weight | | 3.93 ± 0.37 | 4.12 ± 0.21 | 4.14 ± 0.34 | 4.20 ± 0.23 |
| Weight (kg) | 1-6 weeks | 4.09 ± 0.34^A^ | 4.32 ± 0.26^B^ | 4.25 ± 0.49^B^ | 4.13 ± 0.16^A^ |
|  | 7-10 weeks | 3.98 ± 0.34 | 4.10 ± 0.27 | 4.17 ± 0.34 | 4.18 ± 0.12 |
| Feed intake (g/d) | 1-6 weeks | 128.49 ± 13.22^A^ | 126.54 ± 18.28^A^ | 121.69 ± 16.21^Ba^ | 119.21 ± 18.98^Ba^ |
|  | 7-10 weeks | 113.11 ± 1.37^A^ | 106.13 ± 6.62^B^ | 103.82 ± 5.72^Bb^ | 104.23 ± 4.32^Bb^ |

Values within the same column not sharing a common uppercase superscript letter (a. b. c. etc.) differ significantly (P < 0.05) but values within each row not sharing a common superscript letter (A. B. C. etc.) are significantly different (P < 0.05).
